# Machine Learning and Explainable AI for Breast Cancer Patient Prioritization: An Intelligent Decision-Support Framework

**DOI:** 10.3390/bioengineering13091044

**Published:** 2026-09-08

**Authors:** Fabián Silva-Aravena, Jenny Morales, Hugo Núñez Delafuente, César González-Zúñiga

**Affiliations:** 1 Facultad de Ciencias Sociales y Económicas, Universidad Católica del Maule, Avenida San Miguel 3605, Talca 3460000, Chile; jmoralesb@ucm.cl (J.M.); henunez@ucm.cl (H.N.D.); 2 Grupo APEMIN, Facultad de Administración y Negocios, Universidad Autónoma de Chile, Talca 3460000, Chile; cesar.gonzalez@uautonoma.cl

**Keywords:** breast cancer risk, explainable artificial intelligence, patient prioritization, machine learning, decision support systems

## Abstract

Breast cancer continues to represent a major global health burden, highlighting the need for effective approaches to risk stratification and clinical decision support. Conventional methods, including the Breast Imaging Reporting and Data System (BI-RADS) and histopathological classifications, primarily rely on clinical assessments and may not fully account for relevant demographic and behavioral characteristics. To overcome these limitations, we present an integrated framework combining K-Means clustering, Random Forest classification, and Explainable Artificial Intelligence (XAI) to support breast cancer risk stratification and patient prioritization. The proposed methodology uses clustering to stratify patients into low-, medium-, and high-risk groups, followed by supervised machine learning to reproduce the cluster-derived risk categories, achieving an accuracy of 98%. To enhance interpretability, Local Interpretable Model-Agnostic Explanations (LIME) are incorporated to identify the variables that most strongly influence individual classifications, including body mass index (BMI), breastfeeding practices, and maternal age. By integrating multiple dimensions of patient information, the framework provides a more comprehensive characterization of risk while increasing the transparency of the decision-making process. Its relatively simple and scalable architecture also facilitates potential implementation in healthcare environments with limited resources. Simulation experiments further provide a proof-of-concept evaluation of the proposed prioritization approach. Compared with random patient selection, the strategy achieved a substantially higher average severity score (1.66 vs. 0.92) and prioritized 4.3 times more high-risk patients. These findings suggest that the proposed framework can serve as an intelligent decision-support tool for prioritizing breast cancer patients and improving resource allocation when healthcare capacity is constrained.

## 1. Introduction

According to [1,2], breast cancer remains one of the most prevalent and lethal diseases worldwide, posing significant challenges for early detection, risk stratification, and personalized patient care. In resource-limited healthcare systems, especially in developing countries, efficient allocation of medical resources and targeted intervention strategies are imperative to improve patient outcomes [3,4,5]. This study aims to address these challenges by proposing a novel hybrid framework for data-driven risk stratification and prediction in breast cancer patients, using advances in machine learning (ML) and XAI [6,7,8].

Traditional risk assessment methods, such as BIRADS and histopathological classifications, have been instrumental in the diagnosis and evaluation of breast cancer [9,10]. Although these tools provide valuable clinical information, they often rely on subjective interpretations and are limited to narrowly defined clinical parameters, excluding critical demographic and behavioral factors that influence patient outcomes [11]. The advent of ML techniques has introduced powerful predictive capabilities to this field, offering the potential to process and analyze heterogeneous data sets [12]. However, the lack of integration between ML models and broader patient data, coupled with the opacity of many ML algorithms, hinders their adoption in clinical workflows. Few studies [7,13] have effectively combined unsupervised clustering with supervised learning techniques to achieve a comprehensive and interpretable risk stratification approach.

Existing methodologies often operate in isolation, focusing on grouping patients into homogeneous groups or predicting individual risk levels using supervised models [14]. This siloed approach overlooks the potential synergies of integrating these techniques into a unified framework. Moreover, the absence of interpretability in many predictive models, commonly referred to as the “black-box” problem, further limits their clinical utility [15,16]. XAI methods, such as LIME, have recently emerged as critical tools to address this challenge by providing transparency into the predictions of the model (see, e.g., [7]). However, the application of XAI in the stratification of breast cancer risk remains underexplored, leaving a significant gap in translating the ML results into actionable clinical strategies [17].

To overcome these gaps, our work integrates clustering, supervised learning, and XAI techniques into a cohesive framework, aiming to address the dual challenges of predictive accuracy and model interpretability. Specifically, we combine K-Means clustering with a random forest classifier to stratify patients into low-, medium-, and high-risk clusters, while using LIME to provide patient-level explanations for model predictions. This framework is designed to empower clinicians with actionable insights, improve the prioritization of breast cancer patients, and optimize resource allocation in constrained healthcare settings.

The main contribution of our work is the development of a hybrid methodology that integrates unsupervised clustering, supervised learning, and explainable artificial intelligence into a comprehensive patient prioritization framework. Unlike studies that apply these techniques independently, our approach explicitly links risk stratification, prediction, and interpretability to support clinical decision-making. Importantly, this study does not address cancer detection or prognosis; instead, it focuses on risk-based prioritization within confirmed breast cancer cases to support efficient allocation of healthcare resources. Accordingly, our proposed framework should not be interpreted as a cancer detection or computer-aided diagnosis system, but as a decision-support tool for clinical management in settings where demand exceeds available capacity.

The remainder of our paper is structured as follows. Section 2 reviews the existing literature on breast cancer risk stratification and the application of ML techniques, highlighting current gaps. Section 3 details our proposed methodology, including data pre-processing, clustering, supervised learning, and LIME integration. Section 4 presents the experimental results and evaluates the performance and interpretability of our framework. Section 5 discusses the implications of the findings and Section 6 concludes with key insights and directions for future research.

## 2. Related Works

Breast cancer remains a leading cause of morbidity and mortality worldwide, underscoring the critical need for effective risk stratification tools in clinical decision-making [1,18,19]. Traditional methodologies (e.g., BIRADS and histopathological classifications) have been instrumental in assessing risk [9,10,20]. However, these methods often lack objectivity, have a narrow focus, and depend on certain clinical data ignoring behavioral and demographic factors that may substantially influence patient outcomes [11]. Recent advances in computational techniques offer new opportunities to improve risk stratification by integrating diverse data sets into unified frameworks [21].

The introduction of ML in healthcare has revolutionized risk prediction, enabling data-driven insights from large and heterogeneous datasets [22,23,24]. Previous studies [25,26,27] have shown that supervised learning models, including Random Forest (RF), Support Vector Machines (SVM), and Gradient Boosting Classifiers, have demonstrated strong predictive performance in the diagnosis and prognosis of breast cancer. Despite their efficacy, these models are often criticized for their lack of interpretability, which hinders their acceptance and implementation in clinical workflows [15,16]. Furthermore, clustering algorithms, such as K-Means, have shown the potential to group patients into homogeneous risk categories [28,29].

XAI techniques, such as LIME and Shapley Additive Explanations (SHAP), have emerged to address the interpretability challenge posed by complex ML models [7,30,31]. By elucidating the factors driving the predictions, XAI fosters trust and transparency in the output of the model, enabling clinicians to derive actionable insights [30]. The integration of XAI with predictive models and clustering techniques could help bridge the gap between predictive performance and interpretability, offering a transformative approach to clinical decision-making [32,33].

Existing methodologies tend to operate in silos, focusing on clustering or supervised learning without exploiting the potential synergies of combining these techniques [34,35,36]. In addition, most models overlook biopsychosocial factors such as lifestyle behaviors, emotional states, and demographic variables, which are critical to a comprehensive understanding of patient risk [37,38,39,40,41,42,43]. The “black-box” nature of many ML models further exacerbates this problem, creating barriers to their clinical adoption [15].

Recent studies [21,44] have emphasized the promise of hybrid frameworks that integrate unsupervised and supervised learning techniques. For instance, combining K-Means clustering with supervised models has been shown to improve risk stratification [34,45]. However, these studies often fail to incorporate XAI methodologies, which are essential to make such models interpretable and clinically applicable. Inclusion of tools such as LIME provides an additional layer of transparency, enabling clinicians to understand predictions at an individual level and make better-informed decisions [46].

Recent research demonstrates the rapid evolution of XAI in healthcare. Studies such as [47] have compared explainable AI approaches, including LIME, SHAP, and Grad-CAM, highlighting their different capabilities for providing local and global model explanations. In parallel, emerging architectures based on Kolmogorov–Arnold representations are exploring interpretability at the model-design level rather than exclusively through post-hoc explanations. For example, ref. [48] recently investigated hybrid AI architectures incorporating Kolmogorov–Arnold networks, illustrating this broader trend toward interpretable model structures. Although these developments are promising, our work focuses specifically on post-hoc patient-level explanations of a RF model within a risk-prioritization framework.

Recent studies demonstrate the increasing integration of predictive modeling and XAI in breast cancer applications. In [49], the authors combined ML prediction with explainability techniques, while recent approaches such as [50] combine RF and SHAP to provide predictive modeling and feature-level explanations. These studies primarily address breast cancer detection or classification, whereas our framework addresses a different decision problem: relative stratification and prioritization among already confirmed breast cancer cases.

### Justification of the Chosen Method

In light of the existing literature, several critical gaps persist in breast cancer risk stratification that our proposed methodology directly addresses:

- Traditional approaches often focus exclusively on clustering patients into risk groups or predicting risk levels using supervised learning. These disconnected strategies overlook the advantages of combining the two. Our methodology bridges this gap by integrating K-Means clustering with a RF classification, creating a comprehensive framework for risk stratification and prediction.
- Many predictive models operate as “black-box” systems, limiting their acceptance in clinical environments. By incorporating LIME, we ensure that our predictions are transparent and interpretable, fostering trust among clinicians and enabling actionable patient-level insights.
- Current models often emphasize clinical factors while ignoring biopsychosocial dimensions such as lifestyle behaviors and demographic characteristics. Our methodology includes these variables, enhancing the robustness and completeness of the risk stratification process.
- High-dimensional data sets can lead to inefficiencies and overfitting in ML models. Through robust feature selection techniques, we optimize the computational efficiency of our framework without compromising predictive accuracy.

In summary, our hybrid methodology integrates clustering, prediction, and interpretability into a unified framework, directly addressing the limitations of existing approaches. By combining reproducible cluster-based stratification with model interpretability, we provide a decision-support approach for breast cancer patient prioritization. This approach supports structured patient prioritization while also aligning with the resource-constrained realities of many healthcare systems, emphasizing the need for actionable and transparent decision-making tools.

## 3. Methodology

We designed a comprehensive methodology to prioritize patients diagnosed with breast cancer using their individual risk profiles. Using exploratory data analysis and K-Means clustering, we stratified patients into low, medium, and high risk groups, followed by a Ranfom Forest classifier to accurately predict risk levels and highlight critical predictors at the patient level using the interpretability offered by LIME.

**Figure 1:**
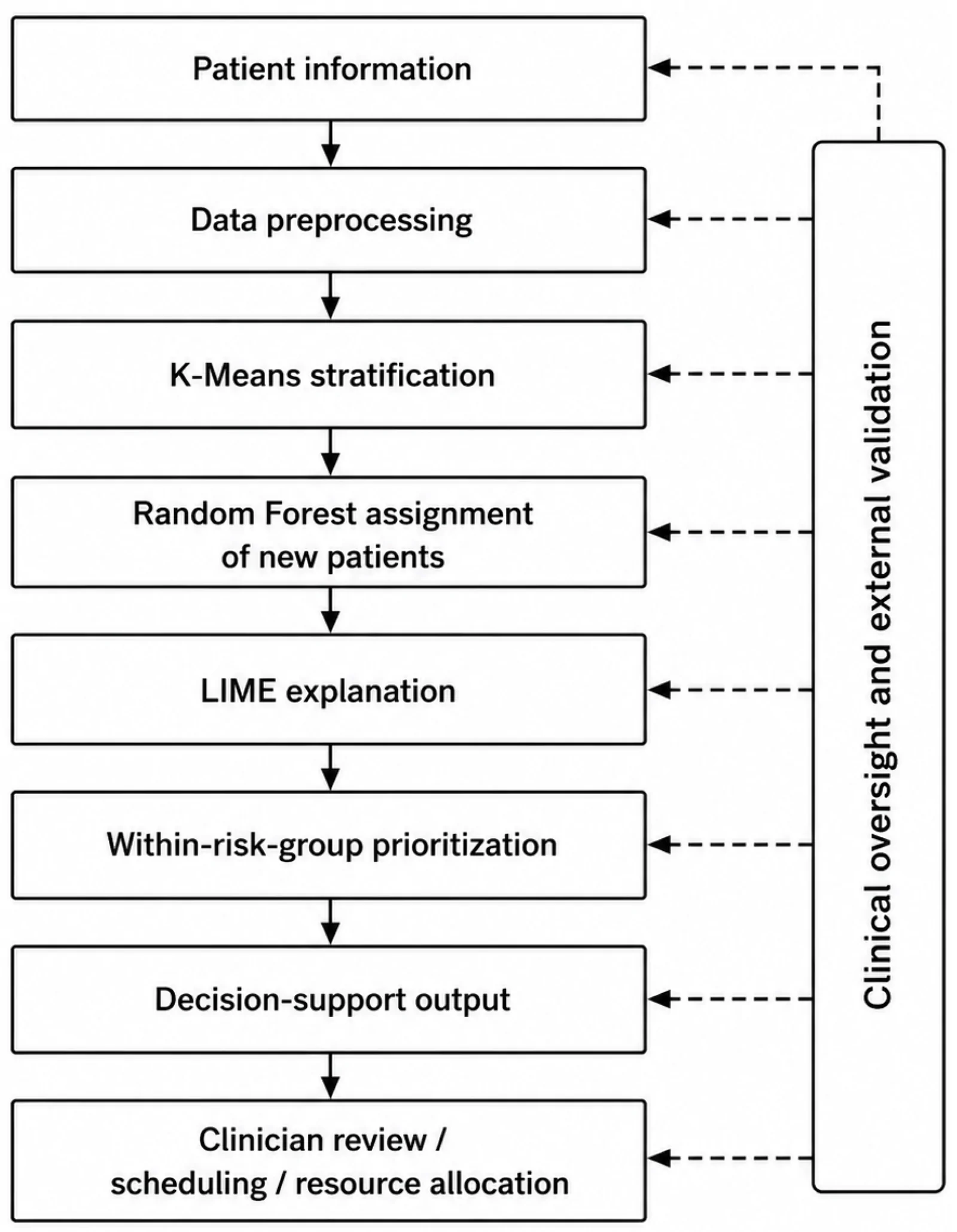
Proposed decision-support workflow and its integration with clinical management.

Figure 1 summarizes our proposed workflow for decision support, which integrates data preprocessing, stratification, supervised allocation, explainability, and prioritization. Our framework is designed to support clinical decision-making, not to replace professional judgment.

### 3.1. Background on Breast Cancer Data

The data in this study contain risk factors for breast cancer from 1697 Cuban women, collected in consultations at the Comandante Manuel Fajardo Clinical-Surgical University Hospital in Havana, Cuba [51]. For our analysis, we have considered only anonymous cases of women with confirmed breast cancer, which are 1160 characterized by 21 variables, covering key clinical, demographic, and behavioral characteristics, such as age, family history of breast cancer, histological classification, mammography findings (BIRADS), and lifestyle factors, such as alcohol and tobacco consumption. The data set includes only confirmed breast cancer cases; therefore, our objective is relative risk stratification and prioritization rather than incidence prediction.

We emphasize that our objective is not to define an external clinical ground truth, but to construct a relative prioritization framework within confirmed breast cancer cases. Therefore, the clustering stage does not replace clinical gold standards; rather, it serves as a multidimensional segmentation mechanism to organize patients according to shared risk profiles.

The initial data set consisted of clinical, demographic, and behavioral explanatory variables. We excluded the patient identifier and the outcome label (breast cancer diagnosis). To improve transparency and reproducibility, we provide details of the variables and their selection status after preprocessing in Table 1. This allows us to identify the variables that contribute to the final risk stratification and prioritization process.

Variables such as BMI, duration of breastfeeding, and maternal age represent explanatory risk factors rather than outcome variables. Consequently, we incorporate them as predictors that contribute to multidimensional stratification, not as target labels.

This data set has ethical approvals to comply with patient privacy regulations. The data set is structured and enriched to support predictive modeling, clustering, and optimization analyzes, providing valuable information for public health strategies and personalized patient care in Cuba.

### 3.2. Data Treatment and Preprocessing

We applied a standard preprocessing pipeline to ensure data quality and comparability prior to clustering and predictive modeling. Specifically, we removed observations or variables with excessive missing values or duplicates, numerically encoded categorical variables, standardized continuous features, and selected relevant variables based on their linear association with the target outcome. This standardization prevents variables with larger numerical scales from disproportionately influencing the Euclidean distances used in the subsequent clustering and prioritization stages.

We excluded observations or variables when the proportion of missing values exceeded a predefined threshold (λ=0.30). We follow established recommendations to reduce bias, according to authors such as [52,53]. We transform categorical variables using ordinal coding to ensure numerical compatibility with the learning algorithms. We standardized continuous variables to zero mean and unit variance to avoid scale-related distortions.

Finally, all variables that exceeded a specific threshold (|ρ|>0.3) were retained by Pearson’s correlation analysis (as suggested in [54]) for features to be included in the model. Among the options, we chose the Pearson correlation because it is a clear and interpretable method and it is reproducible in structured epidemiological datasets. Following correlation analysis, we limited the inclusion of variables higher than this threshold for modeling, as described in Table 1. This act reduced redundancy and noise, preserving clinically relevant insights and enhancing the robustness and interpretability of the model.

We acknowledge that other feature selection techniques (mutual information criteria; recursive feature elimination; embedded tree-based selection strategies) can give us different variable subsets and may have a significant impact on the model performance. A comparative review of feature-selection methods and structured ablation analyzes is a promising direction for future work on model robustness evaluation.

### 3.3. Cluster Analysis

We group the observations into three clusters (*low*, *medium*, and *high*) using the K-Means algorithm. Our objective is to minimize the sum of squared distances within each cluster ($C_{j}$):

(1) $$\begin{array}{c} J_{k}=\min_{q_{1},q_{2},\ldots ,q_{k}}\sum_{j=1}^{k}\sum_{p\in C_{j}}{∥p-q_{j}∥}^{2}, \end{array}$$

where p represents a data point in multidimensional space, $q_{j}$ is the centroid of cluster $C_{j}$, and *k* is the number of clusters. For this analysis, we set k=3, which corresponds to three risk groups: *low*, *medium*, and *high*.

To determine the optimal number of clusters, we employ the Elbow Method [55], evaluating the sum of squares in the cluster ($J_{k}$) for various values of *k*. The distinct inflection point observed at k=3 reflects the diminishing returns from the addition of additional clusters. This choice aligns with common clinical strategies to stratify medical risks (see, e.g., [56,57,58]).

### 3.4. Supervised Predictive Model

Next, we outline the construction and implementation of our supervised predictive model. We describe the model training process, the calculation of feature importance, and the techniques employed to ensure robustness and interpretability.

#### 3.4.1. Model Training

We implement a random forest classifier model (F) as an ensemble learning method comprising *T* decision trees ($\psi_{t}$) [59,60]. The final prediction for an input x is denoted as $\hat{y}\left(x\right)$, which represents the predicted class label for x. This prediction is determined through majority voting across the ensemble, mathematically expressed as

(2) $$\begin{array}{c} \hat{y}\left(x\right)={\mathrm{argmax}}_{o}\frac{1}{T}\sum_{t=1}^{T}\delta \left(\psi_{t}\left(x\right)=o\right), \end{array}$$

where $\hat{y}\left(x\right)$ is the predicted class label for the input x, *o* is a candidate class, and $\delta (\psi_{t}\left(x\right),o)$ equals 1 if $\psi_{t}\left(x\right)=o$ and 0 otherwise.

In our implementation, we construct each decision tree using bootstrap sampling and introduce randomness by selecting a subset of features at each split. This design improves diversity among trees, reducing the risk of overfitting and enhancing the model’s generalization performance. By aggregating predictions from multiple decision trees, our approach is particularly effective for managing complex, high-dimensional datasets and exhibits robustness to noise and variability in the data.

#### 3.4.2. Feature Importance

We calculate the importance of a variable $V_{j}$ as the sum of impurity reductions (ΔI) at all nodes where $V_{j}$ is used within Random Forest. This is defined as follows.

(3) $$\begin{array}{c} \mathrm{Importance}\left(V_{j}\right)=\sum_{t=1}^{T}\sum_{\nu \in N_{t}}\Delta I_{\nu }\left(V_{j}\right), \end{array}$$

where $N_{t}$ is the set of nodes in the tree *t*, $\Delta I_{\nu }\left(V_{j}\right)$ corresponds to the reduction in impurity at node ν when divided into variable $V_{j}$.

By adding impurity reductions to all trees and nodes, we quantify the contribution of each variable to the overall predictive performance of the model. This approach allows us to identify the most influential characteristics, which are critical to understanding the underlying structure of the data and the decision-making process of the Random Forest.

In our implementation, we construct each decision tree using bootstrap sampling, introducing randomness by selecting a subset of features at each split. This design improves diversity among trees, reduces overfitting, and improves generalization performance. By aggregating predictions from multiple decision trees, we enable the model to effectively handle heterogeneous high-dimensional datasets.

We emphasize that the supervised model is trained using cluster-derived labels rather than an independent clinical endpoint. Consequently, the Random Forest does not infer clinical risk; instead, we use it to generalize the multidimensional stratification structure defined by K-Means. Although this introduces a structural dependency between the unsupervised and supervised stages, we position the supervised component as a scalability mechanism that allows the cluster-based prioritization logic to be consistently applied to new incoming patients. Accordingly, its objective is not to establish an independent clinical risk predictor, but to evaluate whether the resulting stratification rule can be transferred to previously unseen observations. The predictive metrics reported below therefore quantify agreement with the data-driven stratification rather than clinical validity against an external gold standard.

### 3.5. Model Validation

To evaluate the performance of the supervised model, we randomly partition the data set into training subsets (80%) and testing (20%). No patient overlap occurred between training and testing data. We assessed classification performance using standard multi-class evaluation metrics, including precision, recall, F1-score, and overall accuracy, allowing us to evaluate the model’s ability to correctly discriminate between risk categories while balancing sensitivity and predictive reliability in a prioritization context.

This validation procedure reduces direct leakage between training and testing during the supervised classification stage. However, since the target categories originate from the grouping procedure, it does not constitute independent clinical validation. External validation would require risk categories or clinical outcomes established independently by clinicians, or longitudinal follow-up of patients.

### 3.6. Interpretability with LIME

To explain individual predictions, we employ LIME (L) as a method for local interpretability [61,62]. The process is as follows.

We selected LIME because the explainability component of our framework focuses on patient-specific local explanations, rather than a global interpretation of the model. Its independent nature allows us to intuitively present the contribution of individual patient characteristics to a specific RF prediction. LIME should not be considered superior to other XAI approaches. Its local surrogacy approach can be sensitive to disturbance sampling and neighborhood definition, especially in nonlinear environments.

1. We generate a synthetic data set (S) around the input sample x, perturbing the original data while maintaining its local structure. This data set S consists of instances similar to x but with slight variations.
2. We train a local interpretable model (G) using S by minimizing the following objective function:
  (4) $$\begin{array}{c} G=\underset{g\in H}{\mathrm{argmin}}\left[L\left(F,g,\pi_{x}\right)+\Omega \left(g\right)\right], \end{array}$$
  where $L(F,g,\pi_{x})$ is the loss function measuring the discrepancy between the predictions of the global model F and the local surrogate model *g*, weighted by the proximity function $\pi_{x}$, $\pi_{x}$ is a proximity weighting function that assigns higher weights to synthetic data points closer to x, Ω(g) is a regularization term that penalizes the complexity of the local model *g*, ensuring interpretability.

By approximating the behavior of the global model F within the neighborhood of a specific input x, we are able to generate interpretable explanations. This approach allows us to identify the key features that influence individual predictions, providing insight into the decision-making process of the model.

### 3.7. Prioritization Within Clusters

The centroid ($q_{j}$) represents the central point of the cluster $C_{j}$, as defined in Section 3.3. We calculate the centroid as the mean of all points assigned to that cluster. To measure the relative position of a patient *A* within their cluster, we compute the Euclidean distance to the centroid of the cluster [63]. We selected the Euclidean distance because of its simplicity, interpretability, and direct consistency with the distance criterion used by K-Means:

(5) $$\begin{array}{c} D\left(A,q_{j}\right)=\sqrt{\sum_{n=1}^{N}{\left(A_{n}-q_{j,n}\right)}^{2}}, \end{array}$$

where *N* is the total number of dimensions in the data space, $A_{n}$ is the coordinate of patient *A* in the *n*-th dimension and $q_{j,n}$ is the *n*-th coordinate of the centroid for the cluster *j*.

To refine intra-cluster prioritization and ensure comparability, we normalize the distances within each cluster to a range between 0 and 1:

(6) $$\begin{array}{c} \mathrm{Normalized}\;\mathrm{Distance}=\frac{D\left(A,q_{j}\right)-\min\left(D\right)}{\max(D)-\min(D)}, \end{array}$$

where min(D) and max(D) are the minimum and maximum distances in cluster $C_{j}$, respectively.

We assign weights ($w_{j}$) to each cluster ($w_{1}=1$, $w_{2}=2$, $w_{3}=3$) for *low*, *medium*, and *high-risk* groups, respectively, ensuring that:

(7) $$\begin{array}{c} \max\left(\mathrm{Normalized}\;\mathrm{Distance}\right)\cdot w_{j}<\min\left(\mathrm{Normalized}\;\mathrm{Distance}\right)\cdot w_{j+1}. \end{array}$$

The weighted prioritization score for each patient is then computed as follows:

(8) $$\begin{array}{c} \mathrm{Weighted}\;\mathrm{Prioritization}=\mathrm{Normalized}\;\mathrm{Distance}\times w_{j}. \end{array}$$

Finally, we classify patients according to their assigned risk group, followed by their weighted prioritization score within each cluster. This process ensures targeted prioritization, with high-risk patients closer to the centroid given precedence in clinical interventions. Patients with higher scores are prioritized before others within the same risk group.

This means that the prioritization we propose is based on distance and is applied only after supervised risk classification. This ensures that the classification within the cluster reflects both the similarity structure and the predictive risk.

## 4. Results

We present the results of our methodology, which effectively classifies patients into risk levels (*low*, *medium*, *high*) using K-Means clustering and evaluates their prioritization through random forest predictions. In addition, we employ LIME to uncover the key features influencing individual risk predictions.

### 4.1. Elbow Method Analysis for Optimal Clustering

**Figure 2:**
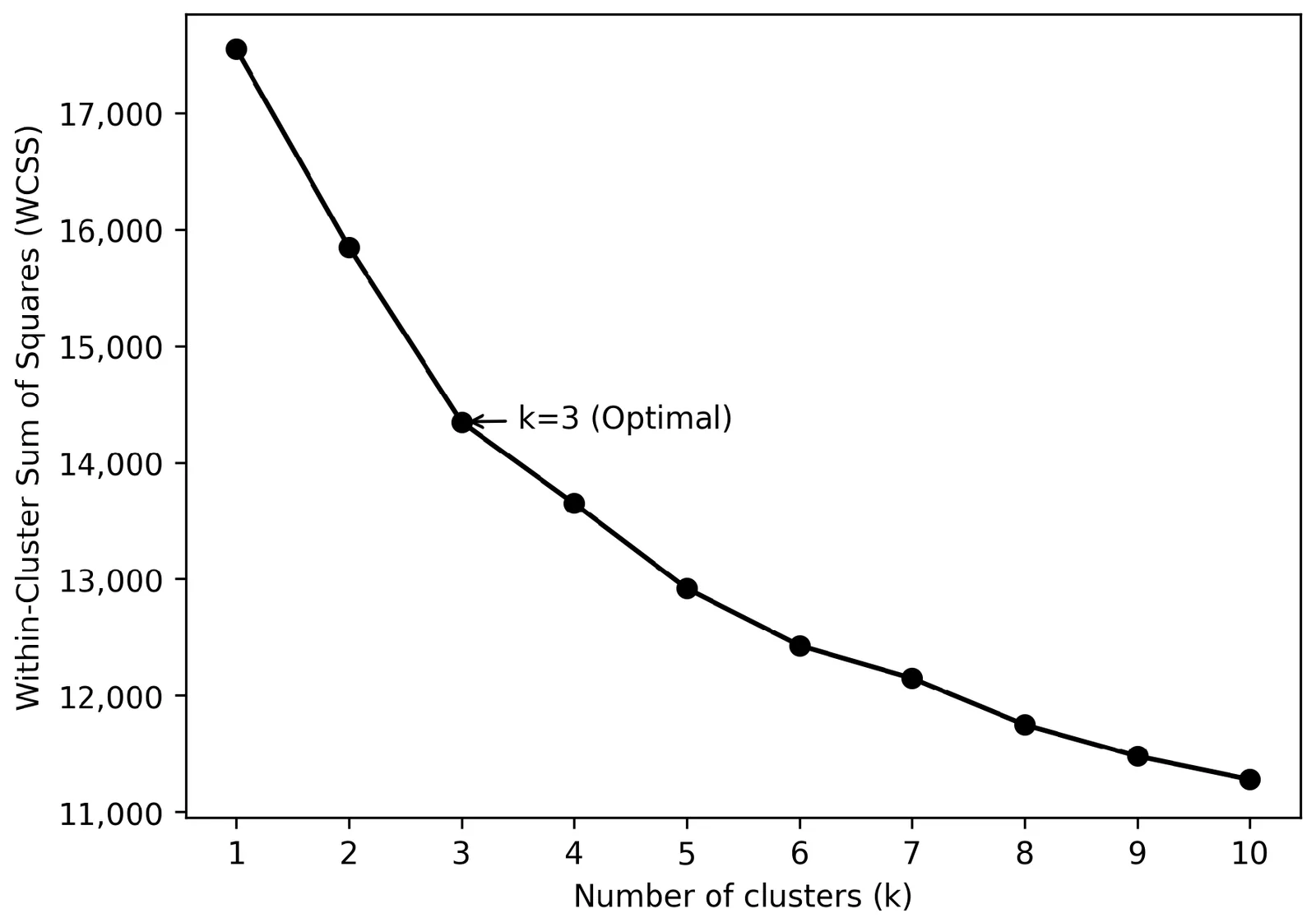
Elbow method for optimal K.

The Elbow Method graph (Figure 2) illustrates the *Within-Cluster Sum of Squares* (WCSS) as a function of the number of clusters *k*. We quantify WCSS as the sum of squared distances between individual data points and their respective cluster centroids. As expected, we observe that the WCSS decreases monotonically as the number of clusters increases, a behavior attributed to the reduction in the average distance between data points and their nearest centroids. A distinct “elbow” point emerges at k=3, indicating the optimal number of clusters. By selecting this value, we avoid overfitting while ensuring that the resulting clusters remain interpretable and meaningful.

**Figure 3:**
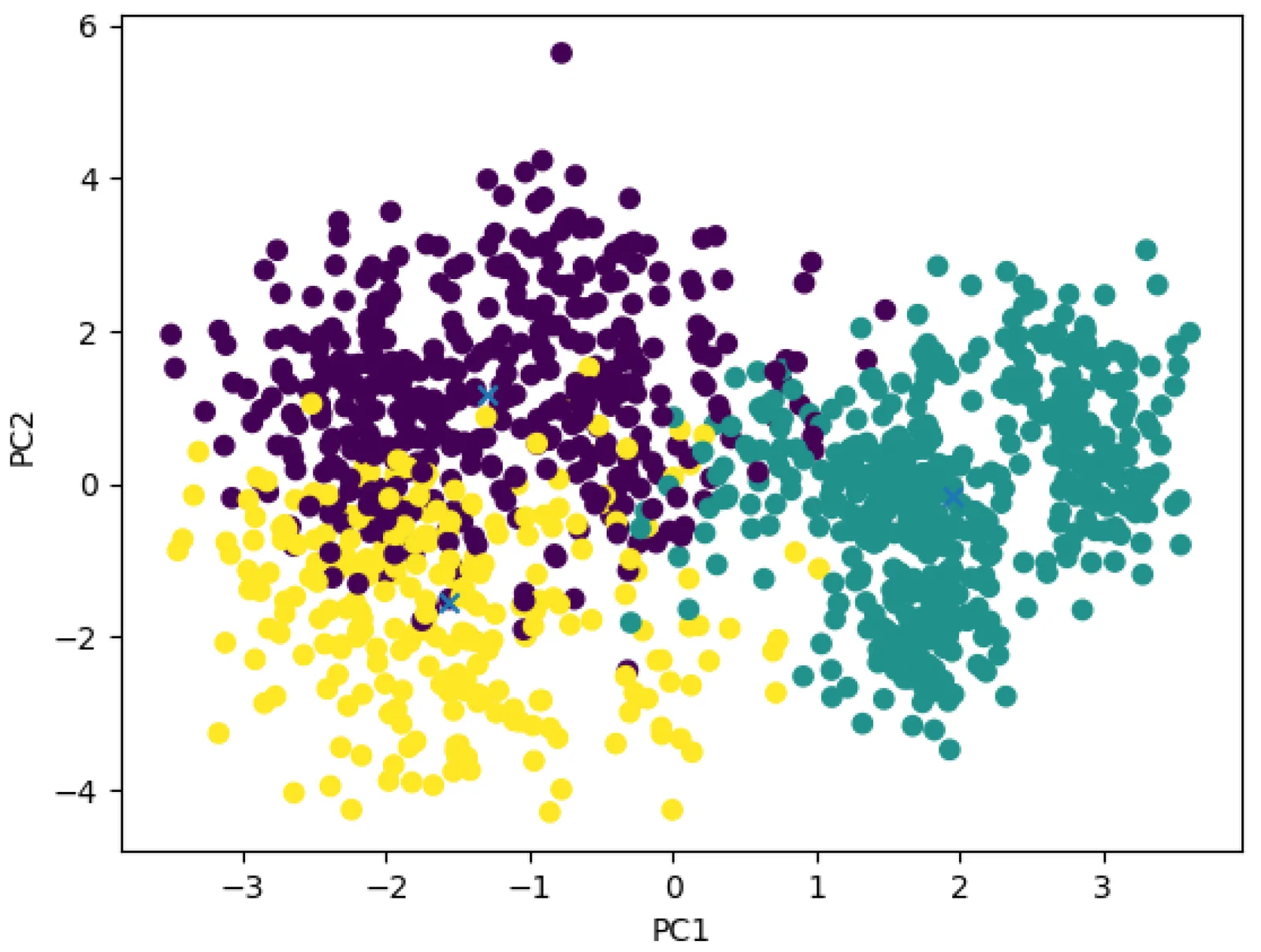
Visualization of patient clusters projected onto the first two principal components (PC1 and PC2).

Figure 3 illustrates the spatial separation of the three risk groups obtained by clustering K-Means, projected onto the first two principal components. Although we used PCA for visualization purposes, we highlight the differentiation between low-, medium-, and high-risk groups, which supports the clinical interpretability of the clustering stage.

### 4.2. Supervised Model Performance Evaluation

We evaluated the performance of our Random Forest model for predicting breast cancer risk levels, categorized into three clusters: *low* (Cluster 0), *medium* (Cluster 1), and *high* (Cluster 2). The general accuracy achieved by the model is 98%. Table 2 summarizes the precision, recall, and F1 score for each risk category.

The results show robust performance across all risk categories. For low-risk patients, the model achieves a precision of 99% and a recall of 100%. Patients with medium risk exhibit the highest precision and recall, at 98%, indicating the strong ability of the model to differentiate medium risk cases. High-risk patients achieve balanced performance with precision and recall at 98% and 97%, respectively. These results indicate that the RF algorithm accurately reproduces the cluster-derived stratification in unseen test observations. We emphasize that the 98% accuracy represents agreement with risk categories based on data generated by the clustering stage and should not be interpreted as independently validated clinical predictive accuracy.

The confusion matrix in Table 3 provides additional insight into the predictions of the model by showing the distribution of the true and predicted risk levels.

In summary, our RF algorithm achieved consistent agreement with the cluster-derived labels across all three risk strata. This result supports its use as a mechanism for assigning new observations to the previously identified stratification structure. However, independent clinical validation is still required before interpreting these categories as externally validated clinical risk levels.

To contextualize the predictive and explainability results of our work, we present Table 4, which summarizes two recent XAI-based breast cancer studies and contrasts their main methodological features with our proposed approach. Since these studies address different clinical tasks and use distinct datasets and validation designs, we suggest interpreting this comparison only within its context.

Unlike these recent approaches, which primarily address detection or malignancy classification, our framework focuses on the relative stratification and prioritization of patients with already confirmed breast cancer.

### 4.3. Local Interpretability Analysis of Patients

We selected two representative patients (Patients 13 and 26) for illustrative purposes to demonstrate the LIME-based interpretability mechanism and to understand the key characteristics influencing their risk classifications. The weighted prioritization score was included as a clinically derived variable rather than a direct model outcome, allowing us to assess how stratification and prioritization jointly influence explanations at the individual level. In practical clinical deployment, borderline or clinically ambiguous cases would represent the most relevant candidates for LIME-based analysis, as interpretability is particularly valuable when clinical decisions require additional transparency.

**Figure 4:**
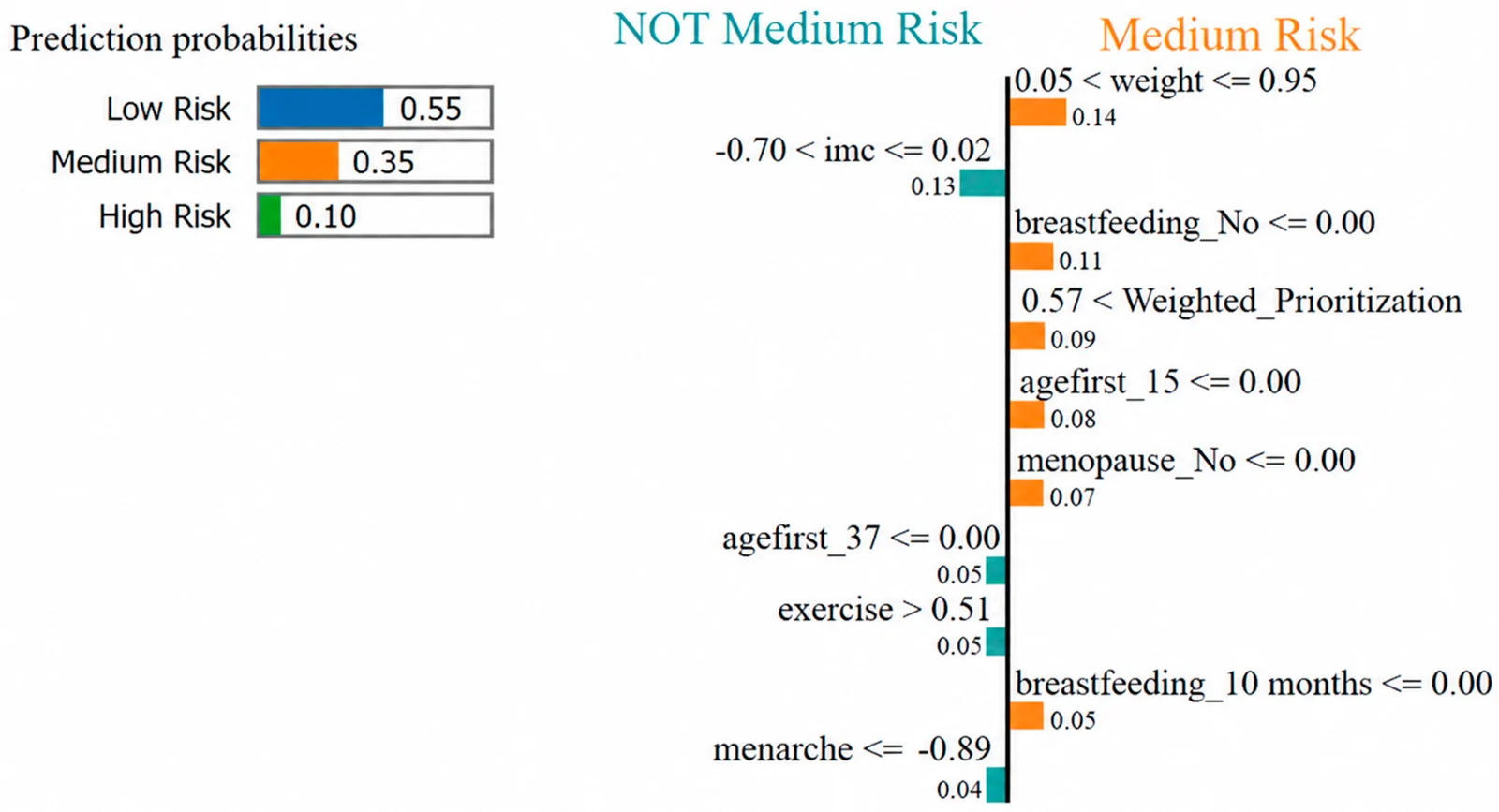
LIME analysis for Patient 13.

For Patient 13, in Figure 4, the model predicts a higher probability of belonging to the low-risk group (55%), followed by the medium risk (35%). Features such as low BMI and early menarche push the prediction toward low risk, while weight, absence of breastfeeding, and weighted prioritization contribute to the medium risk classification.

**Figure 5:**
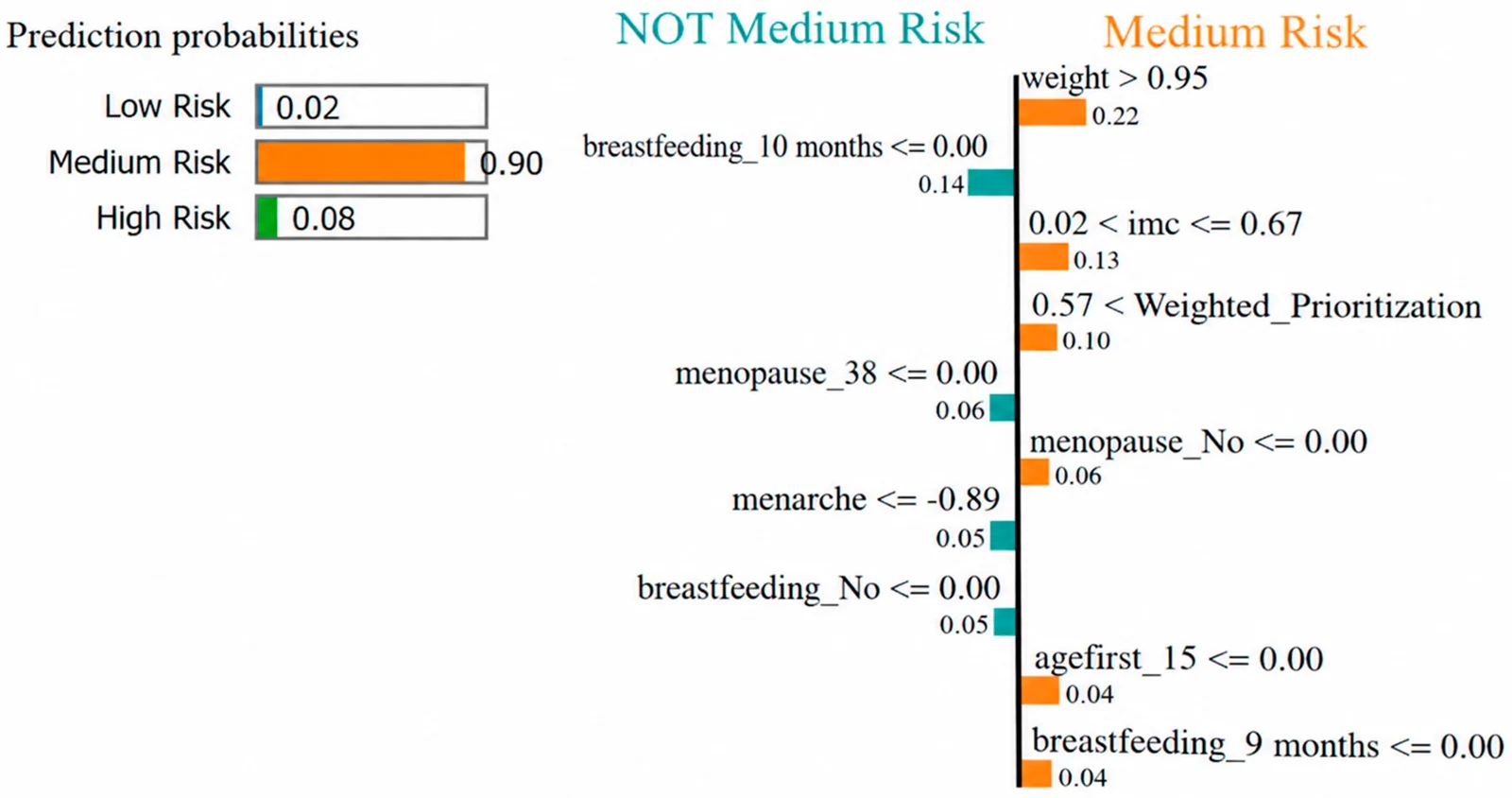
LIME analysis for Patient 26.

Patient 26 exhibits a strong probability of being in the medium-risk group (90%), with minimal probabilities for low (2%) and high risk (8%). Key contributing factors include high weight, extended duration of breastfeeding, and weighted prioritization, which reinforce the prediction of medium risk (see Figure 5).

These local interpretations allow us to identify specific features that influence individual predictions, offering actionable insights for clinical decision-making and intervention prioritization.

The patient-level explanations also illustrate that some cases may exhibit characteristics associated with more than one risk stratum. For example, Patient 13 shows a less differentiated prediction, with contributions supporting both low- and medium-risk assignment. In such cases, LIME provides transparency regarding the features driving the classification rather than concealing this uncertainty behind a single predicted label.

### 4.4. Prioritization Results by Risk Group

In our analysis, we prioritize patients within each risk group (low, medium, and high) using Weighted Prioritization scores. This approach ensures consistent rankings within each group, emphasizing clear differentiation of individuals based on their proximity to the cluster centroid. To illustrate the prioritization process, we present three tables (Table 5, Table 6 and Table 7), one for each risk cluster. This methodology effectively identifies patients that require immediate attention while maintaining interpretability and clinical relevance at all risk levels.

### 4.5. Proof-of-Concept Evaluation of the Prioritization Strategy

To validate the effectiveness of our prioritization strategy, we performed a simulation comparing its results with a baseline random selection scenario. The objective is to assess the ability of the framework to prioritize high-risk patients with breast cancer efficiently and equitably.

We used a data set of 300 breast cancer patients, categorized into low, medium and high risk groups. Each patient was assigned attributes such as age, BMI, comorbidities, and a severity score indicative of their clinical condition. Severity scores were proportionally adjusted based on risk levels, ensuring that higher-risk groups exhibited greater severity. Two selection methods were simulated: (1) random selection of 60 patients, and (2) prioritization using our strategy, which ranks patients by risk scores and severity within each risk cluster.

In the random selection method, patients were chosen without regard to risk or severity. In contrast, our prioritized method allocated resources starting with high-risk patients, followed by medium- and low-risk groups, ensuring that severity scores dictated the order of selection within each group. The two scenarios we compared based on the average severity of the selected patients and the number of high-risk patients included. Table 8 summarizes the findings:

The simulation results provide a proof-of-concept evaluation of the operational behavior of our prioritization strategy. The average severity score of the patients selected using the framework (1.66) exceeded that of the random selection method (0.92), illustrating its ability to prioritize patients assigned to higher-risk strata. Furthermore, the prioritization framework selected all 60 high-risk patients, compared to only 14 high-risk patients included in the random scenario. These findings illustrate the potential operational value of the proposed strategy for patient prioritization under capacity constraints, while external clinical validation remains necessary.

Our findings provide a proof-of-concept evaluation of the prioritization logic in the simulated resource-constrained scenario. They demonstrate the expected operational behavior of the classification strategy we propose but should not be interpreted as evidence of clinical efficacy.

## 5. Discussion

We present a unified framework that integrates clustering, supervised learning, and explainable artificial intelligence to improve the stratification and prioritization of breast cancer patients. Rather than addressing diagnosis or treatment selection, our approach focuses on risk-based prioritization by ranking patients within clinically significant risk strata. In this context, our strategy supports decisions related to follow-up, scheduling, and allocation of resources, particularly in hospitals characterized by high demand and limited capacity.

A key strength of our framework lies in the seamless combination of K-Means clustering and Random Forest classification. Using clustering to group patients and supervised learning to assign new observations to the cluster-derived risk strata, we combine descriptive and predictive capabilities. This dual approach generates interpretable and actionable insights, making it particularly valuable for clinical decision-making. Additionally, the incorporation of LIME enhances transparency by explaining individual predictions, addressing a major limitation in many ML models.

Another notable advantage is the use of a multidimensional data set that includes clinical, demographic, and behavioral variables. This holistic approach facilitates a nuanced understanding of risk factors. Furthermore, the simplicity and scalability of the proposed framework make it suitable for deployment in resource-constrained healthcare settings, where computational efficiency and interpretability are paramount. Compared with recent XAI-based breast cancer studies, our main contribution is the integration of stratification, supervised assignment, patient-level explainability, and explicit prioritization within a single decision-support framework.

Despite these strengths, we acknowledge several limitations of our framework.

(1) The supervised labels are generated from the K-Means clustering stage, rather than from independently established clinical outcomes. Our RF model assesses the reproducibility of the data-driven stratification structure, rather than independent prediction of clinical risk. While our test-data assessment measures performance on unseen observations, it does not eliminate this structural dependency. Therefore, the 98% accuracy we present should not be interpreted as clinical validation. Retrospective or prospective external evaluation using clinician-defined risk categories or longitudinal outcomes is needed to establish the clinical validity and generalizability of the framework. Furthermore, we did not establish any formal threshold for defining overlapping or ambiguous cases; their definition and clinical validation should involve clinical expert assessment in future studies;
(2) We employ the Euclidean distance for our prioritization metric, which is a geometric approximation of multidimensional similarity within each cluster. Although this measure is common to clustering applications, it is currently not a clinically valid severity score. Alternative distance measures, such as the Mahalanobis distance, which accounts for covariance structure, may offer improved clinical sensitivity and should be explored in future work;
(3) We acknowledge that additional dimensionality reduction techniques, such as Principal Component Analysis (PCA), could further improve scalability in high-dimensional datasets. Furthermore, we focus on a single study population and this may restrict generalization of the results to other demographic or geographical groups. Comparisons with recent studies should also be interpreted cautiously because they use different datasets and clinical endpoints; future evaluation on common independently labeled datasets and external populations would enable more direct benchmarking and assessment of generalizability.
(4) We did not assess the stratification of the risk derived with respect to long-term clinical outcomes (e.g., recurrence, death, or worsening). This means that risks should be interpreted as relative prioritization strata rather than prognostic categories. External validation using longitudinal or follow-up data represents an important future step in enhancing clinical applicability.
(5) We use LIME to provide intuitive, patient-specific explanations; however, its local surrogate approximation can be sensitive to perturbation sampling and neighborhood definition. SHAP provides a theoretically grounded alternative based on Shapley values and can offer both local and global feature attribution. A comparative assessment, including explanation stability, represents an important direction for future work.

Future research should prioritize further broadening the dataset to cover different populations and exploring temporal modeling approaches to enable a more dynamic risk assessment. Further comparisons between supervised models and deeper robustness tests, such as structured ablation studies, are still key areas of research to improve the empirical evaluation of our proposed framework. In addition, such developments could further improve the sensitivity and applicability of our method to different clinical contexts.

In conclusion, our framework effectively integrates clustering, prediction, and interpretability, addressing critical gaps in breast cancer risk stratification. By aligning this methodological strategy with clinical applicability, our approach provides a solid foundation for advancing data-driven tools to support patient prioritization and healthcare resource allocation.

## 6. Conclusions

In this study, we develop a unified framework that integrates clustering, supervised learning, and XAI to improve breast cancer risk stratification. By combining K-Means clustering with a Random Forest classifier and incorporating LIME for interpretability, we propose a scalable and interpretable framework for patient stratification and prioritization.

Our framework achieved an accuracy of 98% in reproducing the cluster-derived stratification on unseen test observations. Inclusion of clinical, demographic, and behavioral variables allowed a detailed understanding of patient risk profiles, with factors such as BMI, breastfeeding practices, and maternal age identified as influential features in individual predictions. The use of LIME further enhances model interpretability by providing patient-specific explanations of individual predictions.

In our proof-of-concept simulation, we further examined the operational behavior of the proposed prioritization strategy under capacity constraints. Compared with random selection, we observed that our framework favored patients assigned to higher-risk strata, supporting its potential use for structured patient prioritization when healthcare resources are limited. We interpret these findings as evidence of the operational behavior of the proposed strategy rather than clinical effectiveness, which will require validation in independent clinical settings.

The adaptability of this approach across various healthcare systems is a key strength. By tailoring the framework to specific populations and resource constraints, it can support decision-making in various clinical contexts. This flexibility is particularly valuable in low- and middle-income countries, where resource-efficient tools are essential to optimize care and resource allocation.

In conclusion, in this work, we present an interpretable, data-driven prioritization framework that translates machine learning output into actionable decision support for breast cancer care. By explicitly focusing on risk-based prioritization rather than disease detection, our proposed approach aligns with real-world clinical workflows and resource management needs. In addition, the framework is designed to be scalable and adaptable, providing a solid foundation for future data-driven healthcare tools designed to support patient prioritization and optimize resource utilization.

## Figures and Tables

**Table 1:** Profile of explanatory variables and correlation-based feature selection.

Variable Category	Variable	Type	Selected Features
Demographic	Age	Continuous	Yes
Demographic	Race	Categorical	No
Demographic	Year of diagnosis	Continuous	No
Reproductive	Menarche (age at first menstruation)	Continuous	Yes
Reproductive	Menopause age	Categorical	Yes
Reproductive	Age at first birth (Agefirst)	Continuous	Yes
Reproductive	Number of children	Ordinal	No
Reproductive	Breastfeeding duration	Continuous	Yes
Family history	First-degree relatives with BC (Nrelbc)	Categorical	Yes
Clinical	Number of biopsies	Continuous	No
Clinical	Atypical hyperplasia	Binary	Yes
Clinical	Histological classification	Ordinal	Yes
Clinical	BIRADS category	Ordinal	Yes
Anthropometric	Body Mass Index (BMI)	Continuous	Yes
Anthropometric	Weight	Continuous	Yes
Behavioral	Physical activity (Exercise)	Ordinal	No
Behavioral	Alcohol consumption	Binary	No
Behavioral	Tobacco consumption	Binary	No
Psychosocial	Emotional predisposition	Binary	No
Psychosocial	Depressive symptoms	Binary	No
Other clinical	Allergies	Categorical	No

**Table 2:** Classification Report: Precision, Recall, F1-Score, and Support for Breast Cancer Risk Prediction.

Risk Level	Precision	Recall	F1-Score	Support
Low	0.99	1.00	0.99	80
Medium	0.98	0.98	0.98	92
High	0.98	0.97	0.97	60
Accuracy	0.98			
Macro Avg	0.98	0.98	0.98	232
Weighted Avg	0.98	0.98	0.98	232

**Table 3:** Confusion Matrix: True vs. Predicted Risk Levels.

True	Predicted		
	Low	Medium	High
Low	80	0	0
Medium	1	90	1
High	0	2	58

**Table 4:** Comparison with recent XAI-based breast cancer approaches.

Study	Clinical Task	Model	XAI Approach	Performance	Explicit Patient Prioritization
Arravalli et al. (2025) [49]	Breast cancer detection/benign–malignant classification	Multiple ML models; Random Forest showed the strongest individual performance	SHAP, LIME, ELI5, Anchor, and QLattice	Accuracy = 89%; F1-score = 84%; AUC = 0.96	No
Akben and Yumrutaş (2025) [64]	Breast tumor malignancy pre-screening	Decision Tree and RUSBoost ensemble	SHAP/TreeSHAP	Accuracy ≈ 91.7%	No
**Proposed framework**	**Relative stratification of confirmed breast cancer cases**	**K-Means + Random Forest**	**LIME**	Accuracy = 98% *	**Yes**

* Reported performance values are not directly comparable due to differences in datasets and study designs. The 98% accuracy of our framework reflects agreement with cluster-derived labels, not independent clinical validation.

**Table 5:** Top 10 Prioritized Patients for Cluster 0 (Low Risk).

Pacient	Distance to Centroid	Normalized Distance	Weighted Prioritization
1	5.161	0.977	0.977
2	5.011	0.932	0.932
3	4.791	0.867	0.867
4	4.645	0.824	0.824
5	4.411	0.755	0.755
6	4.398	0.751	0.751
7	4.347	0.736	0.736
8	4.247	0.707	0.707
9	4.238	0.704	0.704
10	4.207	0.695	0.695

**Table 6:** Top 10 Prioritized Patients for Cluster 1 (Medium Risk).

Pacient	Distance to Centroid	Normalized Distance	Weighted Prioritization
1	4.397	0.751	1.502
2	4.387	0.748	1.496
3	4.178	0.686	1.373
4	4.157	0.680	1.360
5	4.154	0.679	1.359
6	4.128	0.671	1.343
7	4.087	0.659	1.319
8	4.049	0.648	1.296
9	4.041	0.646	1.291
10	3.960	0.622	1.244

**Table 7:** Top 10 Prioritized Patients for Cluster 2 (High Risk).

Pacient	Distance to Centroid	Normalized Distance	Weighted Prioritization
1	5.240	1.000	3.000
2	4.715	0.845	2.535
3	4.691	0.838	2.513
4	4.451	0.767	2.300
5	4.369	0.743	2.228
6	4.325	0.730	2.189
7	4.141	0.675	2.026
8	4.139	0.675	2.024
9	4.053	0.649	1.948
10	4.048	0.648	1.944

**Table 8:** Comparison of Random and Prioritized Selection Scenarios.

Scenario	Average Severity of Selected Patients	Total High-Risk Patients Selected
Random Selection	0.92	14
Prioritized Selection	1.66	60

## Data Availability

The dataset used in this study is publicly available at [https://data.mendeley.com/datasets/7jhddnpz2p/1, accessed on 29 August 2026]. The complete code required to reproduce the computational experiments is available at [https://github.com/FabianSilvaA/Breast-Cancer-Prioritization-XAI, accessed on 29 August 2026].
